# SAMHD1 Functions and Human Diseases

**DOI:** 10.3390/v12040382

**Published:** 2020-03-31

**Authors:** Si’Ana A. Coggins, Bijan Mahboubi, Raymond F. Schinazi, Baek Kim

**Affiliations:** 1 Department of Pediatrics, School of Medicine, Emory University, Atlanta, GA 30032, USA; s.a.coggins@emory.edu (S.A.C.); bijan.mahboubi@emory.edu (B.M.); rschina@emory.edu (R.F.S.); 2 Center for Drug Discovery, Children’s Healthcare of Atlanta, Atlanta, GA 30032, USA

**Keywords:** SAMHD1, dNTPs, viruses, Aicardi–Goutières syndrome, cancers

## Abstract

Deoxynucleoside triphosphate (dNTP) molecules are essential for the replication and maintenance of genomic information in both cells and a variety of viral pathogens. While the process of dNTP biosynthesis by cellular enzymes, such as ribonucleotide reductase (RNR) and thymidine kinase (TK), has been extensively investigated, a negative regulatory mechanism of dNTP pools was recently found to involve sterile alpha motif (SAM) domain and histidine-aspartate (HD) domain-containing protein 1, SAMHD1. When active, dNTP triphosphohydrolase activity of SAMHD1 degrades dNTPs into their 2′-deoxynucleoside (dN) and triphosphate subparts, steadily depleting intercellular dNTP pools. The differential expression levels and activation states of SAMHD1 in various cell types contributes to unique dNTP pools that either aid (i.e., dividing T cells) or restrict (i.e., nondividing macrophages) viral replication that consumes cellular dNTPs. Genetic mutations in SAMHD1 induce a rare inflammatory encephalopathy called Aicardi–Goutières syndrome (AGS), which phenotypically resembles viral infection. Recent publications have identified diverse roles for SAMHD1 in double-stranded break repair, genome stability, and the replication stress response through interferon signaling. Finally, a series of SAMHD1 mutations were also reported in various cancer cell types while why SAMHD1 is mutated in these cancer cells remains to investigated. Here, we reviewed a series of studies that have begun illuminating the highly diverse roles of SAMHD1 in virology, immunology, and cancer biology.

## 1. SAMHD1 is a dNTPase Comprised of an N-terminal SAM Domain, a Central HD Domain, and C-Terminal Regulatory Domain

Steady-state intercellular dNTP pools are maintained through carefully regulated cellular processes dedicated to the synthesis and degradation of dNTP molecules: While enzymes, such as ribonucleotide reductase (RNR) [1,2,3,4] and thymidine kinase (TK) [5,6], contribute to dNTP biosynthesis, sterile alpha motif (SAM) domain and histidine-aspartate domain (HD)-containing protein 1 (SAMHD1) degrades dNTPs into 2′-deoxynucleosides (dNs) and triphosphates by its dNTP triphosphohydrolase (dNTPase) activity. Enzymes involved in dNTP biosynthesis are upregulated during S phase to accommodate DNA replication [7,8,9]. Conversely, SAMHD1 expression remains relatively consistent throughout the cell cycle [10], only accumulating in cultures upon starvation-induced quiescence [9]. The dNTPase activity of SAMHD1 is, instead, regulated post-translationally.

Human SAMHD1 is a 65-kDa protein comprised of three structural domains (Figure 2): (1) An N-terminus SAM domain preceded by a nuclear localization sequence (^11^KRPR^14^), (2) a central, catalytic HD domain containing the conserved metal-coordinating histidine and aspartic acid residues essential for dNTPase function [11], and (3) a C-terminus regulatory domain harboring both T592, a residue that undergoes phosphorylation by the cyclin A2/CDK complex during S phase [12,13,14], and two cyclin-binding motifs (^451^RXL^453^ and ^620^LF^621^) [15,16]. X-ray structures of the HD domain have revealed that SAMHD1 is active as a dNTPase when in a tetrameric oligomerization state and further studies illuminated the method of tetramerization to involve the occupation of two-A1 and two-A2 allosteric sites as well as the presence of a metal ion within the HD domain active site [17,18]. Apo-SAMHD1, however, primarily exists in inactive monomeric and dimeric forms [19].

During tetramerization, positive cofactors dGTP/GTP first bind within A1 allosteric sites to form inactive dimers [20]. Once both A1 allosteric sites are occupied, any dNTP molecule can bind to the A2 allosteric sites, driving the tetramerization of SAMHD1 [21]. Binding of a nucleotide in the A2 allosteric site stabilizes the dimerization of dimers; however, the extent of cooperative binding depends on which dNTP occupies this site [21]. An active tetramer, then, is stabilized by four allosteric sites. Each allosteric site contacts three monomers in the structure and contains a dGTP/GTP-Mg^2+^-dNTP bridge [22]. Additional metal ions are chelated by the His167-His206-Asp207-Asp311 quartet in order to correctly orient and polarize the dNTP substrate in the catalytic sites of each monomer. An in-line nucleophilic attack of the α-phosphate then hydrolyzes a dNTP into one 2′-deoxynucleoside and a triphosphate. It has been suggested that active tetramers can exist in the cell long after dNTPs have been depleted below the activation threshold (~10 µM) [23,24,25], a feature of SAMHD1 that would be essential for maintaining the nanomolar-range dNTP concentrations found in resting CD4+ T cell and macrophages [26].

The cellular availability of GTP, which is coupled with its capacity to form more stable and active tetramers than dGTP upon binding to the A1 allosteric sites [27], highlights GTP as the primary SAMHD1 activator in vivo [20,27,28]. Stabilizing interactions in the A2 allosteric site create preferential binding amongst the various dNTPs. The larger purine activators have been reported to create stable base-stacking interactions with the guanidine side chain of R333 while this residue forms a salt bridge with E355 [21]. These interactions are essential for dNTPase function [18], resulting in the preferential binding order of dATP>dGTP>dTTP>dCTP within the A2 site [29]. The catalytic site of SAMHD1 is promiscuous, accommodating the structures of all dNTP substrates (dATP, dGTP, dTTP, dCTP, and dUTP) as well as various dNTP analogs, such as ddNTPs and cytarabine (ara-C) [29,30,31,32]. While the catalytic site indiscriminately binds dNTPs regardless of base modifications, it is restrictive to 2′-OH groups on the ribose moiety, thus negating ribonucleotides as substrates for hydrolysis [20]. In the presence of all four dNTPs, SAMHD1 hydrolyzes the substrates at different rates (dGTP>dCTP>dTTP>dATP) [21,33,34]; however, this order is different when observing hydrolysis in the presence of only one dNTP (dATP>dTTP>dCTP>dGTP) [21]. This points to potential structural crosstalk between the allosteric sites and catalytic site during hydrolysis.

## 2. The dNTPase Activity of SAMHD1 Can be Regulated Via Post-Translational Modifications

SAMHD1 is phosphorylated at T592 (pSAMHD1) by the cyclin A2/CDK complex during S phase and dephosphorylated by PP2A with a B55alpha regulatory subunit (PP2A-B55α) during mitotic exit [10]. The identification of cell cycle-regulated phosphorylated SAMHD1 resulted in the quest to characterize this post-translational modification and its effect on the tetramerization and activation of SAMHD1. While many groups found that dNTP activators dissociate from the A2 allosteric sites of pSAMHD1, leading to tetramer destabilization and subsequent loss of triphosphohydrolase activity [22,25,35,36], there are conflicting reports stating that the dNTPase activity of pSAMHD1 and phosphomimetic mutants T592E and T592D are very similar to wildtype SAMHD1 [37,38,39,40]. Despite the discrepancies surrounding the dNTPase activity of pSAMHD1, phosphorylation of SAMHD1 has also been shown to play a role in HIV-1 restriction—a function that we will further discuss in later sections.

In addition to the phosphorylation of T592, SAMHD1 can also undergo K405 acetylation by arrest-defective protein 1 (ARD1) (Figure 2). Acetylation of K405 is highest in G1, resulting in increased dNTPase activity and decreased intercellular dNTP pools [9,41]. Lee et al. concluded this enhanced dNTPase activity of acetylated SAMHD1 aids in G1/S transition, as elevated dNTP pools delay entry into S phase. The satisfaction of this checkpoint promotes cell cycle progression and supports cancer cell proliferation. SAMHD1 knock out (KO) cells accumulate at G1, supporting this hypothesis [9,42].

While the addition of functional groups to a protein through phosphorylation and acetylation is one form of post-translational modification that regulates SAMHD1 hydrolase activity, reversible oxidation of three critical surface-exposed cysteine residues act as an additional layer of regulation in this process. Positioned near the allosteric nucleotide binding sites, residues C522, C341, and C350 create reversible intramolecular bonds that inhibit tetramerization and/or dNTPase activity (Figure 2). While C522 has been identified to have the highest redox activity, formation of a C341-C350 disulfide bond has been observed in numerous crystal structures (PDB: 3U1N, 4MZ7, 4Q7H, 4QG1, 4QG2, and 4RXO) [17,28,29,33]. The presence and function of these transient interactions is under investigation.

In 2017, the Hollis group found that C341, C350, and C522 could undergo inhibitory oxidation, an event that is prevented if tetramerization precedes exposure to the oxidizing agent [43]. Site-directed mutagenesis generating alanine mutations revealed functional differences between the residues. Mutants C341A and C350A retained WT oxidation sensitivity and thus showed decreased tetramerization and dNTPase activity in oxidizing conditions. Conversely, mutant C522A displayed a loss of oxidation sensitivity compared to WT but did not have any differences in tetramerization or dNTPase activity upon H_2_O_2_ treatment. While C341A and C350A mutants retained the ability to form disulfide linkages, C522A and a C341A/C350A double mutant showed no disulfide species in increased hydrogen peroxide conditions, implying that the disulfide bridge involves the triad of cysteine residues. The study concluded that C522 controls the redox switch by forming disulfide bridges with either C341 or C350. The disulfide bridge between C341 and C350 observed in crystal structures would then be the result of a thio-disulfide exchange reaction that stabilizes the inactive species prior to reduction by cellular reductants and regeneration of the “switch”. A study from the Ivanov group reported similar in vitro activity for serine mutants; however, in vivo work revealed WT-like tetramerization and dNTPase activity for all mutants [44].

A simulation study conducted by the Bhattacharya group found that SAMHD1 monomers are compacted in the tetrameric structure, increasing not only protein stability but also solvent accessibility to the catalytic cores. Simulations revealed that C341S and C522S mutations cause drastic disruptions to the allosteric signaling network that extend to the catalytic site [45]. This is interesting since the Ivanov group found that C522S mutants display WT dNTPase activity despite this disorder. Lastly, a disulfide bridge between C341 and C522 mimicked the stable and solvent-accessible monomer structure found in an assembled tetramer, supporting the hypothesis of an in vivo active monomer that is controlled by a redox switch. Along with phosphorylation of T592, the ability of SAMHD1 to undergo redox transformations has been implicated in its ability to restrict viral infection [44].

## 3. SAMHD1 Protein Contains an NLS and is Expressed in a Variety of Cell Types

As a nuclear localization sequence (NLS)-containing protein [46,47,48] (Figure 2), SAMHD1 has been suspected to maintain strict nuclear localization [9,49,50]. However, many studies have demonstrated that SAMHD1 can be partially cytosolic [46,51,52]—maintaining relatively equal nuclear and cytosolic populations in some cell types [53,54]—and that its localization varies due to the cellular environment. Upon oxidative stress, SAMHD1 was found to migrate from the nucleus to the cytoplasm, only to return back to the nucleus one hour after stimulation [43]. Diffuse SAMHD1 nuclear localization can also punctate towards areas of DNA damage [55,56]. Not only does the localization of SAMHD1 vary but so does its expression level in various tissues [57]. SAMHD1 expression is highest in nondividing macrophages, dendritic cells, and quiescent CD4+ T cells while displaying low expression in activated CD4+ T cells. Interestingly, the Wu group discovered that *SAMHD1* promoter methylation directly correlated with low SAMHD1 expression in CD4+ cell lines, such as Jurkat and Sup-T, while primary CD4+ lymphocytes harboring unmethylated promoters were characterized by elevated SAMHD1 expression [9,58]. Additionally, SAMHD1 translation is impaired by miR-181, a microRNA expressed in CD4+ T cells that binds SAMHD1 mRNA in the 3′-UTR to silence translation [59,60]. These studies revealed novel transcriptional and translational regulation mechanisms governing SAMHD1 expression.

Additionally, SAMHD1 expression may be differentiation dependent: SAMHD1 protein expression is greatly increased in PMA-treated THP-1 cells displaying a nondividing phenotype when compared to untreated dividing populations [12,42,44]. Type I interferon stimulation has been seen to induce SAMHD1 expression in primary monocytes [61,62], microglia [63], astrocytes [63,64], liver cells [50,65], HEK293T, and HeLa [66,67] cells while having no effect on protein expression in CD4+ cells [66], dendritic cells [66], MDDCs [12,66], and MDMs [12,68], information that is well summarized in a 2017 review by Jun Li’s group [69]. It is important to note that SAMHD1 expression levels do not necessarily correlate with its dNTPase activity and cellular dNTP pools. This is because the dNTPase function of SAMHD1 is regulated in several ways as mentioned above. In dividing cells, SAMHD1 has been identified to directly interact with the cyclin A2/CDK complex [12,13,16,70,71], CtIP [55,72], SKP2 [13,14], PP2A-B55α [10], cyclin L2 [73], TRIM21 [74], and various proteins involved in nuclear import [48,54]. Direct binding partners of SAMHD1 in non-cycling cells are still unknown.

## 4. SAMHD1 Restricts HIV-1 Infection in Nondividing Viral Target Cells

**Figure 1:**
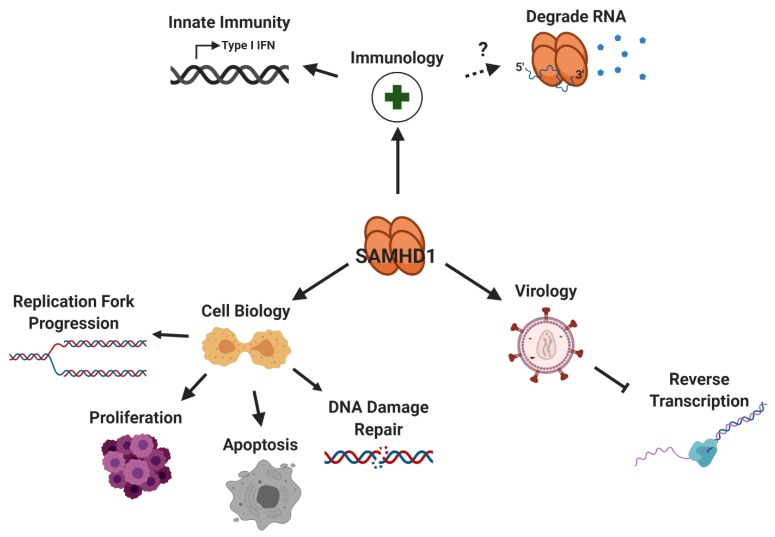
SAMHD1 plays a variety of roles in virology, immunology, and cell biology. The dNTPase activity of SAMHD1 depletes intercellular dNTP pools in macrophages, restricting reverse transcription of HIV-1 in this cell type. Similarly, SAMHD1 has been found to restrict the viral replication of other DNA and RNA viruses. In addition to viral restriction, SAMHD1 facilitates replication fork progression, is implicated in cell proliferation and apoptosis, and is localized to sites of DNA damage. As a negative regulator of IFN I, SAMHD1 is commonly mutated in a disease that phenotypically resembles a congenital viral infection called AGS. The controversial exonuclease activity of SAMHD1 would negatively regulate host innate immunity as aberrant host and viral nucleic acids could serve as potential degradation targets. Figure 1, Figure 2 and Figure 3 were created with BioRender.com.

**Figure 2:**
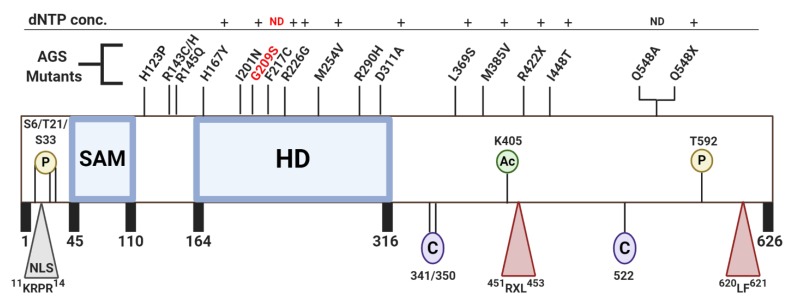
Key SAMHD1 domains, residues, and AGS mutants. SAMHD1 contains an N-terminal SAM domain, central catalytic HD domain, and C-terminus containing an ^451^RXL^453^ and ^620^LF^621^ cyclin-binding motif that is essential for recognition and subsequent phosphorylation of T592 by CDK1/2. SAMHD1 contains a classic NLS sequence (11-KRPR-14), a residue that can undergo acetylation (K405), and at least four residues that can undergo phosphorylation (S6, T21, S33, and well-known T592). Lastly, a triad of surface-exposed, oxidizable cysteines (C341, C350, and C522) have been found to influence protein function. SAMHD1 mutations identified in AGS patients are labeled and distributed along the top of the SAMHD1 schematic. In White et al. (2017), the dNTP concentrations of U937 cells were measured following the transduction of different SAMHD1 AGS mutants. Here, the mutants are summarized to have more (+) or no difference (ND) in dNTP pools relative to wildtype hSAMHD1. SAMHD1 mutants that do not have a symbol assigned represent the population that were not able to be tested. The mutant G209S (highlighted red) represents the only variant found to restrict HIV-1.

**Figure 3:**
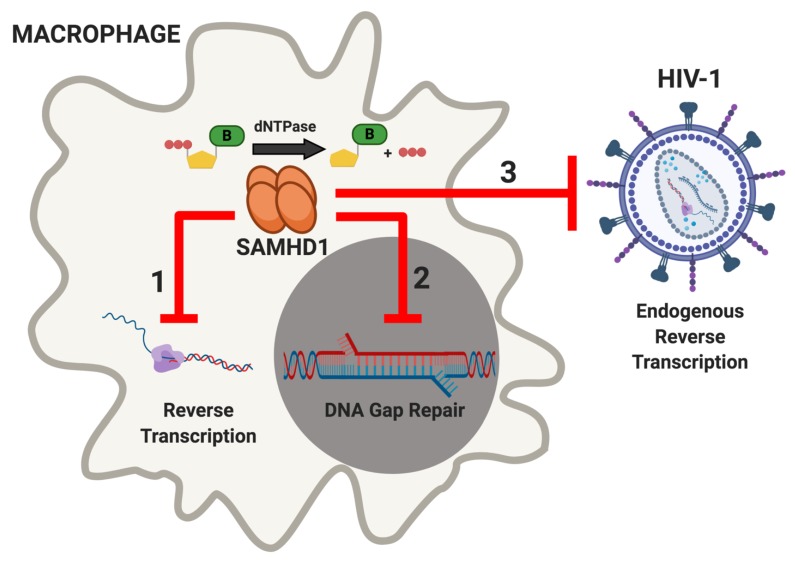
Host SAMHD1 restricts HIV-1 infection within macrophages at the reverse transcription, integration, and ERT steps of viral replication. Tetrameric SAMHD1 (orange), which can reside in the cytosol (beige) and nucleus (dark grey), restricts the HIV-1 lifecycle at three points during viral infection of a macrophage. The SAMHD1-mediated low dNTP pools in macrophages inhibit reverse transcription in the cytosol (red inhibition arrow 1), gap repair within the nucleus (red inhibition arrow 2), and ERT activity occurring extracellularly (red inhibition arrow 3).

The abundance of dNTPs present in the cell at any given time is based on cellular demand and is tightly regulated by several host proteins [75]. Rapidly dividing cells consume dNTPs during DNA replication and logically have a higher abundance of active dNTP biosynthesis machinery [76,77,78], such as RNR and TK [7,79,80]. Conversely, elevated SAMHD1 expression is associated with lower dNTP levels due to its dNTPase activity. The low dNTP pools resulting from the dNTPase activity of SAMHD1 is known to restrict viral replication of some RNA and DNA viruses because host dNTPs are required during the genome replication of these pathogens [81,82] (Figure 1). In human primary macrophages, intercellular dNTPs fall below the *K_m_* of HIV-1 RT [83]. As a result, proviral DNA synthesis by HIV-1 is slowed, as both RNA- and DNA-dependent DNA polymerization kinetics are reduced in the SAMHD1-mediated low dNTP pools of the macrophage. This illustrates that reverse transcription kinetics during the HIV-1 replication cycle is suppressed by the dNTPase activity of host SAMHD1 [84,85]. In low dNTP conditions, HIV-1 RT more readily incorporates non-canonical nucleotides [86,87], displays an elevated strand transfer frequency [88], and increasingly relies on the central polypurine tract for completion of proviral DNA synthesis [89,90]. During HIV-1 integration, partially integrated viral DNA (vDNA) sits between two to three single-stranded DNA gaps until host DNA polymerases use cellular dNTPs to repair the gap [91,92]. The SAMHD1-mediated low dNTP pools in macrophages kinetically delay this step because the 5′-end gap repair is dependent on cellular dNTPs [91]. The low dNTP pools in macrophages have also been shown to reduce endogenous reverse transcription (ERT), the extra-cellular reverse transcription step that partially synthesizes proviral DNAs within cell-free viral particles. Virions produced from dividing cells contain non-selectively packaged dNTPs and experience greater HIV-1 ERT activity, resulting in a more efficient infection in nondividing cells [93]. The effects of low dNTP concentrations on HIV-1 results in an overall attenuation of viral production in macrophages. In summary, there are three steps during the viral replication cycle in which the dNTPase activity of SAMHD1 restricts HIV-1: Reverse transcription, integration, and ERT (Figure 3). 

In addition to the ability of SAMHD1 to restrict viral replication through its dNTPase activity, numerous studies have suggested that phosphorylation of T592 regulates the antiviral activity of SAMHD1 independent of its dNTPase activity. While replacement of T592 with phosphomimetic residues did not alter SAMHD1 dNTPase activity, oligomerization, or localization, T592E and T592D are not able to restrict retroviral infection [38,40]. Interestingly, T592A retained the ability to restrict viral replication [12,40]. Two potential explanations for these observations are either (i) phosphorylation of T592 regulates the interaction of SAMHD1 with an unknown cofactor that is necessary for viral restriction or (ii) phosphorylation of T592 induces a conformational change that abolishes the ability of SAMHD1 to restrict viral replication while maintaining other known functions of the enzyme. Early investigations sought to characterize the potential DNase and RNase activity of SAMHD1, proposing that the dNTPase binds to and degrades vRNA in MDMs and ultimately restricts infection without triggering the innate immune response [94,95]. However, following issues of inconsistency among the reported data [96,97], whether SAMHD1 harbors nuclease activity and whether the putative nuclease activity of SAMHD1 is biologically relevant [98,99] remain unclear.

## 5. Lentiviral Vpx/Vpr Induces the Proteasomal Degradation of SAMHD1

HIV-2 and some SIVs circumvent host SAMHD1 restriction and rapidly replicate even in macrophages by inducing the proteasomal degradation of SAMHD1 through a viral accessory protein called viral protein X (Vpx) [81,82,100,101]. Since Vpx arose from a gene duplication event of viral protein R (Vpr) in ancestral primate lentivirus strains [102,103], some Vpr proteins retain the ability to target SAMHD1 for proteasomal degradation [104,105,106]. Typically, nondividing macrophages harbor intercellular dNTP concentrations of 20–40 nM [26]. However, lentiviral Vpx robustly reduces SAMHD1 protein levels by recruiting SAMHD1 to DCAF1, which in turn complexes with DDB1, cullin4, and the ROC1/RBX1 protein prior to recruitment of the E2 enzyme. The formation of this complex targets SAMHD1 proteasomal degradation [81,82,106,107,108,109]. Direct docking of SAMHD1 to DCAF1 is essential for Vpx-mediated SAMHD1 degradation during human immunodeficiency virus 2 (HIV-2) and simian immunodeficiency virus (SIV) infections via the CRL4-DCAF1 ubiquitin ligase complex [110,111]. The degradation of SAMHD1 results in a transient 10-fold increase of macrophages’ dNTP concentrations, which is above the *K_m_* values of RT proteins, ultimately leading to rapid reverse transcription in macrophages [84,85,112]. Under Vpx-mediated dNTP elevation, all aforementioned dNTPase-related SAMHD1 restriction pathways are attenuated. Accordingly, lentiviruses that are capable of counteracting SAMHD1 have been found to infect macrophages more efficiently than SAMHD1 non-counteracting lentiviruses [85]. The dynamic between host SAMHD1 and viral Vpx effectuated a long-standing host–virus evolutionary arms race, providing evolutionary pressure that has not only driven diversification of the N- and C-termini of SAMHD1 required for recognition by Vpx [113] but also influenced lentiviral reverse transcriptase (RT) evolution and kinetics. RTs originating from lentiviruses that do not counteract host SAMHD1 (SAMHD1 non-counteracting lentiviral RTs) polymerize proviral DNA in macrophage-like dNTP concentrations more efficiently than SAMHD1-counteracting lentiviral RTs [83,114]. This is because SAMHD1 non-counteracting RTs have evolved to execute a faster conformational change step (k_conf_) during the incorporation of a dNTP [115], enabling the virus to circumvent SAMHD1 restriction and replicate in the otherwise restrictive dNTP pools of the macrophage [85]. Interestingly, while HIV-2 and some SIVs have evolved to counteract SAMHD1, FIV and BIV infections do not induce the degradation of their host SAMHD1 proteins [116]. Despite this fact, fSAMHD1 and bSAMHD1 can be targeted for degradation by SIVmac239 Vpx in a process that requires key C-terminus SAMHD1 residues [117], suggesting that primate lentiviruses have evolved to more efficiently counteract SAMHD1 than FIV and BIV. 

## 6. SAMHD1 is a Negative Modulator of the LINE-1 Retrotransposon

Several mechanisms have been shown to regulate long interspersed element 1 (LINE-1), an autonomous retroelement comprising roughly 17% of the human genome [118,119]. TREX1, SAMHD1 and ADAR1 are all negative modulators of LINE-1 and when mutated cause the autoinflammatory disorder Aicardi–Goutières syndrome (AGS) [120,121,122]. Unlike the dNTPase-dependent restriction of certain viruses by SAMHD1, it is reported that SAMHD1 might inhibit LINE-1 retrotransposition through a different mechanism [123]. One mechanism in which SAMHD1 negatively modulates retrotransposition of LINE-1 is by reducing the expression of the ORF2 protein, an enzyme essential during LINE-1 mobilization that has endonuclease and reverse transcriptase activities [121,124,125]. SAMHD1 can also induce cellular stress granule assembly by disrupting the complex formed by eukaryotic initiation factors (eIFs). SAMHD1 was shown to be involved in the phosphorylation of eIF2α and disrupt eIF4A/eIF4G interactions, which promotes large stress granules, sequestering LINE-1 and blocking retrotransposition [123]. Specific SAMHD1 residues have been shown to be important in negatively modulating retrotransposition of the LINE-1. Phosphorylation or bulky residues on position 33 of SAMHD1 were demonstrated to be essential in the inhibition of LINE-1 activity [126]. The importance of the enzymatic activity of SAMHD1 for restricting LINE-1 mobility is still unclear but studies find that restricting LINE-1 retrotransposition is regulated by threonine 592 and relies on a functional HD domain and intact cofactor binding site [127]. There have been numerous conflicting reports regarding the exonuclease activity of SAMHD1 [33,52,97,128,129]; however, the ability of SAMHD1 to modulate intercellular dNTPs and bind nucleic acids hints at its role in innate immunity, a topic we will detail further in subsequent sections. Ribonuclease H2 (RNase H2), another enzyme that when mutated can result in AGS, is found to have opposite effects in relation to LINE-1 activity. The enzymatic activity of RNase H2 has been shown to strongly induce LINE-1 activity, explaining how LINE-1 can retrotranspose without encoding their own RNase H. It is proposed that the RNase H cleavage of the RNA in the RNA:DNA heteroduplexes results in an increase in LINE-1 retrotransposition [130]. These studies demonstrate the different activities of RNase H2, SAMHD1, and LINE-1 and suggests a complex association to AGS pathophysiology. 

## 7. SAMHD1 Restriction of RNA Viruses

While sharing the necessity for dNTPs during the reverse transcription of their viral genomes, various retroviruses have divergent relationships with SAMHD1 (Table 1). Like HIV-1, other lentiviruses, such as equine infectious anemia virus (EIAV) and feline immunodeficiency virus (FIV), are restricted by SAMDH1 in myeloid cells [116,131]. The Landau group found beta-retrovirus Mason Pfizer monkey virus (MPMV) to be sensitive to SAMHD1 restriction whereas alpha-retrovirus Rous sarcoma virus (RSV) was not [131]. Gamma-retrovirus murine leukemia virus (MLV) is unable to infect macrophages and DCs [47,132] despite the detection of early and late reverse transcription products in these cell types [133]. Once identifying that Vpx treatment increased late reverse transcription products but did not lead to the accumulation of 2-LTR products or productive infection, the Cimarelli group concluded that MLV is restricted not only by SAMHD1-mediated low dNTPs in these cell types but also by the lack of vDNA nuclear import [131,133,134,135].

Unlike HIV-2 and some SIVs, MLV and prototype foamy virus (PFV) do not encode accessory proteins that counteract SAMHD1 during the viral cycle [136]. PFV, however, differs from MLV in the fact that its replication in macrophages not restricted by SAMHD1 or aided by Vpx treatment [131]. This insensitivity to target intercellular dNTPs is because reverse transcription occurs late in the PFV lifecycle, resulting in a fraction of mature virions harboring nearly complete vDNA [137,138,139,140]. PFV virions with complete vDNA are able establish a productive infection in monocytes; however, incomplete reverse transcriptase products (RTIs) trigger a STING-dependent innate immune response in this cell type [141]. As a result, T and B lymphocytes prominently support viral replication in vivo, a tropism that possibly contributes to the apathogenic phenotype of PFV [142,143].

Delta-retrovirus human T-lymphotropic virus (HTLV) primarily infects T cells in vivo and can infect macrophages in vitro [144], suggesting the possibility of in vivo T cell-to-macrophage transmission of the virus. Interestingly, even though HTLV infection does not cause the degradation of SAMHD1, cell-to-cell transmission of HTLV is not improved by Vpx treatment [131]. Like PFV, macrophage infection by HTLV triggers an immune response through the cGas/STING pathway; unlike PFV, this host defense response has been proven to be SAMHD1 dependent. SAMHD1-mediated low dNTP pools in macrophages prevent complete HTLV vDNA synthesis, leading to the accumulation of RTIs in the cytosol. Recognition of HTLV RTI by STING induces SAMHD1-mediated apoptosis in myeloid cells, an event that is inhibited by deoxynucleoside (dN) treatment [145].

In addition, SAMHD1 supports viral replication in Zika (ZIKV) and Chikungunya (CHIKV) infections as well. The Missé group recently found that SAMHD1 mRNA and protein is upregulated in ZIKV- and CHIKV-infected patient samples [146]. Degradation of SAMHD1 by Vpx reduces vRNA and virion production for both (+)ssRNA viruses, indicating that SAMHD1 supports viral replication of ZIKV and CHIKV.

## 8. SAMHD1 Restriction of DNA Viruses

The functional role of SAMHD1 as a dNTPase not only affects the synthesis of vDNA during the reverse transcription of retroviral vRNA but also the DNA replication of DNA viruses, such as human papilloma virus (HPV), poxvirus, and various members of the Herpesviridae family. The Morgan group recently discovered that HPV16 is restricted by SAMHD1 in immortalized foreskin keratinocytes. Infection of this cell type by HPV16 resulted in decreased SAMHD1 expression while the absence of SAMHD1 during HPV16 infection resulted in hyperproliferation and increased viral replication [147]. From this, it is possible the virus downregulates SAMHD1 expression just enough to remove viral restriction and enable efficient replication of its vDNA genome while simultaneously retaining the necessary homologous recombination [148,149,150,151] and proliferation regulation [41,152,153,154] functions of SAMHD1 during viral infection [56].

Though not capable of promoting the degradation of SAMHD1, poxviral vaccinia encodes a viral TK from its J2R gene that potentially targets SAMHD1 and other cellular factors for phosphorylation [155,156] to alleviate SAMHD1-dependent viral restriction in MDMs [157]. TK-negative vaccinia infections display reduced in vitro replication in MDMs [157] and decreased virulence in vivo [158,159]. It is still unclear how vaccinia TK promotes viral replication, but viral infection was observed to modestly increase cellular dNTPs. In addition to deploying a viral kinase, vaccinia also encodes its own ribonucleotide reductase enzyme to increase cellular dNTPs during replication of its viral genome [155].

Hepatitis B virus (HBV) is a liver-tropic virus that predominantly targets hepatocytes during infection [160]. As a group VII virus, HBV contains a partial double-stranded relaxed DNA genome (rcDNA) that is replicated through the reverse transcription of a pregenomic-RNA intermediate in the late stages of the replication cycle [160,161,162]. A series of studies have shown that SAMHD1 is not only expressed in HBV target cells [57,163] but also restricts HBV infection in several human liver cell lines [50] as well as Huh7 cells [164]. Initially, it was unclear whether the dNTPase activity of SAMHD1 is required for HBV restriction. In 2014, the Shen group observed the restriction of viral replication by catalytically inactive SAMHD1 [50]; however, later studies concluded that dNTPase activity is required for HBV restriction and that this restriction is abrogated through phosphorylation of T592 [71,164,165]. Interestingly, HBV-infected cells show no changes in SAMHD1 or pSAMHD1, yet they are characterized by increased cellular dNTPs [165].

Studies have shown that HBV viral protein HBx manipulates the cell cycle by stimulating procession through G1 and stalling the cell before S phase [166,167]. Although CDK2 is constitutively expressed throughout the cell cycle [10], the kinase is activated in G1 through cyclin binding and phosphorylation by a CDK-activating kinase (CAK) [168,169,170]. It is proposed, then, that HBV circumvents SAMHD1 restriction by inducing G1 cell arrest, which promotes phosphorylation of SAMHD1 by CDK2, resulting in the elimination of dNTPase activity and elevation of intracellular dNTPs. Similar to other retroviruses, SAMHD1 restriction of HBV appears to act at the reverse transcription step, as SAMHD1 expression exclusively decreases vDNA while vRNA levels and transcription of vRNA remain unaffected [165].

Vaccines are available to prevent HBV infections; however, interferon-alpha (IFN-α) is commonly used to clinically manage chronic HBV cases [171,172,173]. IFN-α increases intercellular SAMHD1 in HBV-infected hepatocytic cell lines [50,165], potentially illuminating a mechanism for the treatment. The role of SAMHD1 in HBV infections is not solely restrictive. A recent study from the McKeating lab highlights the role of SAMHD1 in the DNA repair pathway, reporting that SAMHD1 facilitates the conversion of rcDNA to the viral transcriptional template, cccDNA, early in the HBV replication cycle [174]. Together, these findings reveal pro- and antiviral roles of SAMHD1 in HBV replication.

Like the *Retroviridae* family, the various subtypes of the *Herpesviridae* family have assorted relationships with SAMHD1 during viral replication in the nucleus. SAMHD1 acts as a restriction factor against alpha-herpesvirus HSV-1 in dividing and nondividing THP-1 cells, inhibiting viral replication independent of its T592 phosphorylation state. While HSV-1 infection of MDMs does not induce T592 phosphorylation [175], the lack of discrimination in the SAMHD1 phospho-state is interesting since catalytically inactive SAMHD1 mutant (HD206AA) lost its restriction activity in U937 cells [176]. Like HIV-1, HSV-1 restriction still appears to be linked to the depletion of cellular dNTPs as dN treatment restores viral replication in PMA-treated THP-1 cells. Recent studies found that the CDK4/6 inhibitor palbociclib reduces CDK2 activation, decreases cellular dNTPs, and inhibits HIV and HSV in MDMs [175]. The antiviral activity of the drug was lost upon Vpx treatment, supporting the hypothesis that SAMHD1 restricts HSV-1 through its dNTPase activity. The current evidence of SAMHD1 restriction of HSV-1 highlights the uncertain linkages between SAMHD1 dNTPase activity, restriction potential, and phosphorylation state.

Unlike HPV16, the effects of beta-herpesvirus human cytomegalovirus (HCMV) on SAMHD1 protein expression appear to be cell specific: While HCMV infection of THP-1 cells induces SAMHD1 expression in a mechanism independent of viral gene expression [177], protein levels decrease in HCMV+ MDMs through transcriptional repression and proteasome-dependent mechanisms [178]. Despite the differential effect of HCMV on steady-state SAMHD1 levels, the virus consistently induces phosphorylation of T592 through viral CHPK UL97—in conjunction with cellular CDK1—and restricts viral replication in both cell types [179]. This activity is shared by the viral kinase M97 of murine cytomegalovirus (MCMV) [134,180]. SAMHD1 restriction of HCMV does not appear to be a result of its dNTPase activity, rather SAMHD1 prevents the accumulation of NF-κB on the HCMV MIE gene promoter, inhibiting transcriptional activation and suppressing viral replication [177,181]. In addition to phosphorylating SAMHD1 to alleviate its restriction, UL97 phosphorylates a host of other proteins during viral replication. The kinase activity of UL97 influences IFN receptor signaling [134,182], vDNA replication, virion morphogenesis, nuclear egress, and more [183], making the kinase an exceptional drug target [184]. Interestingly, studies found that latent HCMV infection of CD34+ progenitor cells downregulated expression of various HIV-1 restriction factors, including SAMHD1, APOBEC3G, and Mx2, while upregulating the expression of HIV-1 coreceptors CXCR4 and CCR5 [185]. These findings begin to illuminate a mechanism behind the acceleration of HIV to AIDS in patients previously infected with HCMV [186,187].

Similar to UL97 of HCMV, BGLF4 of gamma-herpes Epstein–Barr virus (EBV) counteracts SAMHD1 through phosphorylation of T592 [179]. Lytic EBV infection in Akata cells caused a decrease of CDK1 protein and did not affect CDK2 protein levels, indicating that T592 phosphorylation is likely the result of both viral BGLF4 and cellular CDK2 activity. Interestingly, the Li group found that the in vitro dTTPase and dCTPase activities of SAMHD1 are attenuated by BGLF4-mediated SAMHD1 phosphorylation while there is no change in dATPase and dGTPase activity. This would likely result in an imbalanced dNTP pool during viral replication of which the implications are unknown. The combinatorial nature of BGLF4 and CDK2 T592 phosphorylation, however, may display a different outcome in vivo. The role of BGLF4 in the EBV replication expands beyond counteraction of SAMHD1 restriction as the viral kinase has been implicated in the following essential viral processes: Phosphorylation of viral and cellular proteins [188], late viral gene transcription [189], nuclear egress [190,191], and vDNA replication [192,193]. The ability of SAMHD1 to restrict both RNA and DNA viruses is summarized in Table 1.

## 9. SAMHD1 Plays a Role in the Innate Immune Response and is Mutated in AGS

AGS is an inherited encephalopathy characterized by the dysregulation of type 1 interferon (IFN) responses and upregulation of interferon-stimulated genes (ISGs) caused by irregularities in the intracellular nucleic acid sensing machinery TREX1, RNASEH2A, RNASEH2B, RNASEH2C, SAMHD1, ADAR1, or IFIH1 [194]. While more than half of the AGS patients exhibit abnormalities in cellular RNase H2 function, a small subset of patients have mutations in the SAMHD1 gene [194,195]. AGS-associated mutations are found throughout the SAMHD1 gene and often lead to defects in the enzyme’s ability to oligomerize and reduce dNTP levels. All SAMHD1 mutants identified in AGS patients lost their ability to block HIV-1 infection except for G209S [196]. The G209S SAMHD1 mutant represents a unique variant, which differentiates the ability of SAMHD1 to restrict HIV-1 and regulate type 1 IFN responses. Exactly how type 1 IFN is upregulated in AGS patients is still uncertain, but the current hypothesis suggests increases in cellular nucleic acids are first detected by pattern recognition receptors (PRRs) and this event initiates an innate immune response [197,198,199,200]. The production of IFNs in AGS patients resembles the immune response patterns for congenital viral infections, further supporting ties between intracellular nucleic acid sensing machinery and interferon responses. Several cell lines have demonstrated an increase in SAMHD1 expression following IFN treatment [67] (Figure 1). While primary macrophages, dendritic cells, and CD4^+^ T-cells do not induce SAMHD1 expression following IFN treatment, these cells experience a decrease in SAMHD1 phosphorylation at the T592 site [12,51,57,66,68]. The relationship between SAMHD1 and the innate immune system is key to understanding more about AGS and HIV-1 restriction.

The ability of HIV-1 to evade detection by the innate immune system and avoid sterilizing immunity contributes to its in vivo persistence [201]. Several studies suggest that the replication of HIV-1 in immune cells falls below a threshold that would trigger an IFN response [17,18], an evolutionary reason why the virus could have lost the gene that encodes Vpx [202]. How SAMHD1 plays a role in triggering an immune response following HIV-1 infection is still controversial. One group reported that downregulated SAMHD1 in HIV-1-infected dendritic cells (DCs) induced an immune response mediated by cGAS and IRF3 activation [203], resulting in DC maturation [204,205]. A more recent report highlights the ability of HIV-1 to suppress toll-like receptor-induced maturation of DCs independent of SAMHD1 [206]. Understanding the role that HIV-1 and SAMHD1 play in regulating the innate immune response is a continued effort with current gaps in the knowledge on the relevant mechanism and pathways.

## 10. The Role of SAMHD1 in DNA Damage Repair and Cell Cycle Regulation

A balanced dNTP pool is essential for maintaining proper DNA replication and genome stability [207]. Imbalances in cellular dNTP pools during DNA replication can induce mismatch incorporations, which result in stalled DNA polymerases [208,209], replication stress [210], reduced replication fidelity [211], and oncogenic transformation [212] (Figure 1). SAMHD1 sits at the crossroads of these cellular processes as its dNTPase activity is crucial for maintaining proper cell cycle progression and replicative behaviors. Dysregulation of dNTP pools in SAMHD1-deficient cells has been associated with increased genome instability and the elevation of IFN-I activation [213]. In general, SAMHD1 KO cells are characterized by increased dNTPs and the accumulation of cells in the G1 phase of the cell cycle [9,42,213]. However, additional features of the KO appear to depend on the cell type. While knock out of SAMHD1 in fibroblasts results in a senescent phenotype [9,213], SAMHD1 KO THP-1 cells display reduced apoptosis and increased proliferation [42]. Keratinocytes infected with HPV16 also display hyperproliferative behavior only in the absence of SAMHD1 [147]. Conversely, the literature shows that overexpression of SAMHD1 is associated with reduced cell proliferation likely due to the depletion of dNTPs necessary to properly replicate genomic DNA [9,56,154,214,215]. These studies implicate SAMHD1 in cell cycle regulation and proliferation control; however, additional insight is needed to clearly delineate the mechanism and extent of SAMHD1 involvement in these processes.

Low dNTP pools are known to induce replication stress and genomic instability through activation of the Rb-E2F pathway [216,217]. Interestingly, abnormal activation of the Rb-E2F pathway can induce double-strand breaks (DSBs) in DNA [218], which are repaired by either nonhomologous end-joining or homologous recombination (HR) [219,220]. Recently, SAMHD1 was found to facilitate HR-DSB repair by recruiting CtIP to the site of DNA damage [55] in order to stimulate the endonuclease activity of MRE11 [72], an essential step of DNA end resection and repair [221,222]. The sequential recruitment of MRE11 by SAMHD1 prevents the accumulation of cytosolic ssDNA, enabling stalled forks to resume replication without triggering an interferon-driven replication stress response. Phosphorylation of SAMHD1 at T592 promotes this function independent of cellular dNTPs pools. Conversely, the Gupta group found that DNA damage induced by topoisomerase inhibitor etoposide (ETO) activates a p53/p21 pathway that downregulates CDK1, resulting in dephosphorylation of T592 and activation of SAMHD1 in MDMs [223]. These studies display the dNTPase-independent and -dependent roles of SAMHD1 in response to differing forms of DNA damage.

## 11. SAMHD1 is Downregulated in Various Cancers and Has Differential Activity against Drug Substrates

Evidence of genome instability brought about through DNA replication stress appears early in human carcinomas [224]. As a negative regulator of both intercellular dNTPs and DNA replication stress, SAMHD1 is directly implicated in several cancers. Cancers, such as chronic lymphocytic leukemia (CLL) [56,225], lung cancer [215], cutaneous T cell lymphoma (CTCL) [226], acute myeloid leukemia [227], and colon cancer [228], are characterized by the downregulation of SAMHD1. Additionally, online databases, such as COSMIC, cBioPortal, and NCI GDC, have recorded over 300 patient-derived SAMHD1 mutations (COSMIC: COSG646673 [229]) [230,231,232]. Cancer-associated mutations, which are scattered throughout the SAMHD1 gene, have been found to modulate in vivo SAMHD1 activity and expression levels [56,228]. Several SAMHD1 cancer-related mutations were shown to have a loss in negatively modulating LINE-1 [233]. This is interesting because SAMHD1 also harbors the ability to bind DNA and RNA through its HD domain, a function in which the SAM domain is dispensable [47,129]. SAMHD1 preferentially binds ssRNA over ssDNA with no sequence specificity but does not bind dsDNA and RNA/DNA heteroduplexes [128,129,234]. Li Wu’s group observed SAMHD1 promotor methylation [226] in an aggressive subtype of CTCL patient samples, indicating transcriptional repression as a SAMHD1 regulation mechanism in CTCL. Despite its downregulation in cancer cells, SAMHD1 expression persists at low levels and interferes with anti-cancer nucleoside analogs, such as ara-C [32]. SAMHD1 can hydrolyze deoxyribonucleoside triphosphates with canonical and modified bases and sugars, including ara-CTP [20,30,235]. The SAMHD1-mediated hydrolysis of nucleoside triphosphate analogues reduces intercellular concentrations of the therapeutic agents and ultimately dampens the anti-cancer efficacy of the treatment. With this, many consider SAMHD1 as a clinical biomarker of ara-C sensitivity in cancers and have suggested the provision of Vpx along with ara-C during treatment to improve efficacy [31,55,236].

Nucleoside reverse transcriptase inhibitors (NRTIs) are one of six classes of antiretroviral drugs and were first approved for their ability to slow acquired immunodeficiency syndrome (AIDS) progression in patients [237]. NRTIs are first phosphorylated to their active 5′-triphosphate state by cellular nucleoside/nucleotide kinases, then compete against dNTPs for incorporation into proviral DNA during reverse transcription [238]. NRTIs act by causing chain termination after they have been incorporated into viral DNA during reverse transcription. NRTIs are especially effective against HIV-1 in myeloid cells due to the presence of SAMHD1, which decreases the levels of dNTPs that can compete with chain terminators during proviral synthesis [239] and thus supports the activation of some NRTIs [94]. The SAMHD1-mediated triphosphate cleavage of NRTIs are performed at a significantly lower rate than cellular dNTPs, allowing this class of drug to efficiently compete for the HIV-1 RT active site [94]. Now that the role of SAMHD1 in anti-HIV-1 drug efficacy is better understood, several nucleotide-based analogues originally not categorized as antiretroviral have been revealed to be potent anti-HIV-1 agents in cells with low dNTP pools. Specifically, two herpes virus-specific drugs and one anti-cancer drug were identified to have enhanced anti-HIV-1 activity in cells with low dNTP pools [239]. These studies present the possibility of repurposing drugs against HIV-1 that were previously overlooked.

## 12. Comparisons between AGS Animal Models

The use of animal models is a longstanding practice in understanding human diseases. Animal models are chosen based on several factors, the most important being physiological similarities. Similar to human SAMHD1 (hSAMHD1), mouse SAMHD1 (mSAMHD1) decreases the dNTP pool and restricts retroviral infection [134]. Alternative splicing results in two mSAMHD1 isoforms that share 72% and 74% sequence homology to hSAMHD1 [240]. Interestingly, while one of the mSAMHD1 isoforms has a regulatory phosphorylation site comparable to T592 in hSAMHD1, the other does not. Only isoform 1 of mSAMHD1 contains a phosphorylation site at residue T634 capable of regulating its antiviral activity while isoform 2 has no comparable site [12,240]. While apo-and holo-hSAMHD1 exists predominantly as a monomer or tetramer, respectively, apo- and holo-mSAMHD1 exists primarily as a dimer and slowly forms tetramers in the presence of non-hydrolyzable nucleotides [241]. This slow progression into a tetramer suggests mSAMHD1 is regulated differently than hSAMHD1 and has a more complex assembly formation.

The generation of SAMHD1-defficient mice has been useful in studying the role of SAMHD1 in the immune response and during viral infection. When mSAMHD1 is knocked out, it triggers upregulation of type I IFN-inducible genes and ISG products. However, the upregulation of IFNs and ISG products in SAMHD1 KO mice was undetectable in most tissues and did not cause the mice to develop neurological impairments or autoimmunity [134,242]. The studies performed in the SAMHD1 KO mice suggests that the loss of mSAMHD1 alone does not induce an equivalent IFN response to what is seen in AGS patients or allow the development of detectable autoimmunity. In contrast, deletion of the AGS-associated genes encoding TREX1 or ADAR1 in mice results in an AGS equivalent autoimmune response as these mice display similar IFN signatures and ISG induction patterns as seen in human AGS patients. Many of the AGS-associated abnormalities in the skin, lung, liver, and brain are also found in the TREX1 KO mice [243,244]. Mice deficient in RNase H2 have an embryonic lethal phenotype due to ribonucleotide accumulation in the DNA causing genome instability [245,246]. Deletion of RNASEH2 in mice does not mimic the AGS pathologies, likely due to the difference in enzymatic activities. RNASEH2 mutations identified in AGS patients only partially reduce the enzymatic activity by affecting the stability and/or catalytic activity, causing an accumulation of ribonucleotide monophosphates in the genomic DNA [247]. Although TREX1 and ADAR1 null mice stand as a suitable animal model for AGS, understanding how each of the nucleic acid sensing machineries play a role in regulating innate immunity is still unclear. The current system in mice used to understand the relationship between loss of SAMHD1 activity and the innate immune response lacks many of the key phenotypic features found in AGS patients. Additional factors required to induce an AGS phenotype in SAMHD1/RNase H2-deficient mice are yet to be discovered.

Although SAMHD1-deficient mice lack the common phenotypic outcomes found in patients suffering from AGS, SAMHD1 morphant zebrafish demonstrate features that suggest they might be a more reliable animal model. Since loss of SAMHD1 results in an innate immune response overlapping pathways associated with viral infections, it is important to note that the antiviral responses (e.g., CHIKV) in zebrafish share similarities with those identified in mammals [248]. The SAMHD1 ortholog identified in zebrafish share 60% amino acid sequence homology to that of hSAMHD1 and also acts as a block against early stages of viral replication. When treated with morpholino antisense oligo targeting the SAMHD1 gene, zebrafish experienced hemorrhage and swelling within the fourth ventricle of the brain. An in-depth analysis of zebrafish reveals that a reduction in SAMHD1 leads to the expression of several genes involved in innate immune responses. The severe brain damage phenotype and IFN induction following the knockdown of the SAMHD1 gene in zebrafish are desirable phenotypes analogous to what is observed in AGS [249]. These studies suggest that the knockdown of the SAMHD1 in zebrafish display a more physiologically accurate depiction of AGS in humans than what was previously observed in SAMHD1 KO mice. Future studies should be performed to establish a stable SAMHD1 mutant in zebrafish that demonstrates the same phenotype observed in the knockdown study.

## 13. Summary and Future Directions

The dNTPase activity of SAMHD1 places this enzyme at the crossroads of various cellular processes, including cell cycle progression and proliferation. Cell cycle progression is rigidly controlled by the fulfillment of cellular checkpoints, some G1/S checkpoints potentially being influenced by the decline of intercellular dNTPs. Many groups have observed the accumulation of cells in G1 due to increased intercellular dNTP pools [9,42,250]. Consistent with this observation, SAMHD1 KO cells display an accumulation in G1, suggesting SAMHD1 has a currently undefined role in cell cycle progression. While RNR-mediated dNTP synthesis peaks in S phase, elevated dNTP pools during G1 have been shown to potentially block the G1/S DNA damage checkpoint and disturb the loading of Cdc45, a component of the preinitiation complex, onto DNA replication origins in yeast [250]. Additionally, cyclin D3 mRNA was found to be downregulated in SAMHD1 KO THP-1 cells relative to control cells. This is interesting since cyclin D3 is known to be involved in G1/S cell cycle progression [42]; however, further investigation is needed to elucidate the potential interplay between cyclin D3 and SAMHD1-mediated cell cycle progression. Conversely to what is seen in SAMHD1 KO cells, the acetylation of K405 in G1 enhances the dNTPase activity of SAMHD1 and promotes transition into S phase. Interestingly, HIV-1 is still capable of infecting macrophages in the G1 phase by upregulating CDK1 and bypassing SAMHD1 restriction [251]. Despite the role of SAMHD1 in cell cycle progression, the precise influence of SAMHD1 on cell proliferation remains unclear. While SAMHD1 KO in THP-1 cells results in a hyperproliferative phenotype, KO of the dNTPase in fibroblasts induced cellular quiescence. The apparent differential sensitivity to aberrations in checkpoint fulfillment might be cell specific due to the dNTPase-independent role of SAMHD1 in the DNA damage response. Treatment of MDMs with ETO has been shown to activate the p53/p21 pathway, a well-known regulator of the G1/S checkpoint that stalls cell cycle progression upon detection of DNA damage, resulting in the dephosphorylation of SAMHD1. ETO treatment of CLL cells, however, results in the recruitment of SAMHD1 to p53 foci and the increase of pSAMHD1 [56]. While both cell types support the recruitment of SAMHD1 to DSBs, the different phospho-states of SAMHD1 may alter its bindings partners, contributing to the ability of the cell to bypass the crucial G1/S DNA damage checkpoint and differentially promote cellular proliferation. As the field continues to uncover novel cellular activities and interactions of SAMHD1 in various cell types, the mechanisms underlying the participation of SAMHD1 in the regulation of the cell cycle and cell proliferation remain to be elucidated.

Loss-of-function mutations in SAMHD1 were found to increase LINE-1 mobilization, an outcome thought to have played a major role in the IFN induction in AGS. The underlying mechanism was proposed to initiate from the accumulation of LINE-1 nucleic acids, which would trigger a cGAS-STING-dependent upregulation of ISGs. However, the proposed mechanism relating LINE-1 and AGS grew complicated once it was discovered that RNase H2 was a positive regulator of LINE-1 restrotransposition and mutations, resulting in AGS severely abrogated LINE-1 mobility. The opposite outcomes of SAMHD1 and RNase H2 on LINE-1 retrotransposition has suggested that RNase H2 might trigger the cGAS-STING pathway in a LINE-1-independent manner. A study performed on mouse embryonic fibroblasts showed that when RNase H2 was knocked out, there was a significant increase in DNA damage and ISGs associated with the cGAS-STING pathway [252]. The accumulation of micronuclei resulting from the DNA damage would be the source for cytoplasmic DNA to activate cGAS. Another hypothesis attempted to reevaluate the established mechanism tying LINE-1 activity and IFN induction by proposing that only LINE-1 by-products and intermediates trigger the type 1 IFN production rather than active retrotransposed elements. This alternative mechanism of activation is further supported by a study that observed the accumulation of LINE-1-associated ssDNA products in TREX1 KO neural cells [253]. Since much of the AGS pathology is centered in the brain, it is of great value to use neural tissue and/or models that express the AGS neurological disorders to examine the nucleic acid machinery and its association to cGAS activation. The impact of LINE-1 on IFN induction seems to only partially explain AGS pathophysiology and might work in conjunction with another IFN initiator.

The ability of SAMHD1 to bind ssDNA and ssRNA molecules has fueled many investigations into its possible exonuclease activity and subsequent impact on SAMHD1-mediated viral restriction. SAMHD1 has displayed metal-dependent 3′→5′ exonuclease activity against ssDNA and ssRNA that requires its HD domain and is independent of its dNTPase activity [128]. While SAMHD1 has been reported to not bind dsDNA and displays weak binding to RNA/DNA heteroduplexes [97,234], the protein is said to preferentially cleave 3′-overhangs of RNAs in RNA/DNA heteroduplexes. An examination of AGS-associated SAMHD1 mutants yielded SAMHD1 variants with selective dNTPase-only (SAMHD1_Q548A_) and RNase-only (SAMHD1D_137N_) activities [52]. Despite the developing characterizations of exonuclease activity, various groups have reported that SAMHD1 is not a nuclease [33,129], explaining the observed DNase and RNase activity as the result of the copurification of SAMHD1 with a nuclease contaminant prior to the conduct of experiments [97]. While the dNTPase function of SAMHD1 is one layer of viral restriction in target cells, many have suggested that SAMHD1 harbors additional dNTPase-independent functions that contribute to its ability to restrict various RNA and DNA viruses. This becomes an area of particular interest when considering the complex links between pSAMHD1, SAMHD1 dNTPase activity, and viral restriction that were highlighted during the extensive characterization of phosphomimetic T592E and T592D mutants. Potential dNTPase-independent mechanisms of viral restriction could involve the differential recruitment of other antiviral restriction factors through its numerous in vivo functional states. It is possible that monomeric/dimeric SAMHD1, tetrameric SAMHD1, pSAMHD1 (and phosphorylation of S6, T21, and S33), acetylation at K405, and the triad of cysteines that modulate the activity and stability of SAMHD1 can all present different protein interfaces to modulate SAMHD1 interaction with cellular proteins involved in viral restriction.

## Figures and Tables

**Table 1:** Different restriction mechanisms of SAMHD1 against RNA and DNA viruses.

Name	Family	Genus	Restricted by SAMHD1 (Y/N) ^†^	Mechanism
HIV-1	Retroviridae	Lentivirus	Y	Incomplete reverse transcription, inhibition of ERT, and restriction of gap repair due to dNTPase activity
EIAV	Retroviridae	Lentivirus	Y	Incomplete reverse transcription due to dNTPase activity
FIV	Retroviridae	Lentivirus	Y	Incomplete reverse transcription due to dNTPase activity
RSV	Retroviridae	α-retrovirus	N	Restricted in MDMs and SAMHD1 KO THP-1s
MPMV	Retroviridae	β-retrovirus	Y	Incomplete reverse transcription due to dNTPase activity
MLV	Retroviridae	γ-retrovirus	Y	Incomplete reverse transcription due to dNTPase activity in conjunction with the SAMHD1-independent lack of vDNA nuclear import
HTLV	Retroviridae	δ-retrovirus	Y	Incomplete reverse transcription due to dNTPase activity pools & RTI-induced apoptosis
PFV	Retroviridae	Spumavirus	N	Late reverse transcription results in nearly complete vDNA in virion
ZIKV	Flaviviridae	Flavivirus	N	SAMHD1 aids viral replication, unknown mechanism
CHIKV	Togaviridae	Alphavirus	N	SAMHD1 aids viral replication, unknown mechanism
HPV16	Papillomaviridae	α-papillomavirus	Y	unknown restriction mechanism countered by viral degradation of SAMHD1
Vaccinia	Poxviridae	Orthopoxvirus	Y	Incomplete DNA replication due to dNTPase activity countered by viral TK and RNR
HBV	Picornaviridae	Hepatovirus	Y	Incomplete reverse transcription of pre-genomic RNA intermediate due to dNTPase activity; aids in rcDNA to cccDNA conversion
HSV-1	Herpesviridae	α-herpesvirus	Y	Incomplete DNA replication due to dNTPase activity
HCMV	Herpesviridae	β-herpesvirus	Y	Prevents NF-κB-dependent transcriptional activation countered by viral kinase UL97
EBV	Herpesviridae	γ-herpesvirus	Y	Incomplete DNA replication due to dNTPase activity countered by viral kinase BGLF4

† Does SAMHD1 restrict this virus? Y = Yes, N = No.
